# Climate‐induced habitat suitability changes intensify fishing impacts on the life history of large yellow croaker (*Larimichthys crocea*)

**DOI:** 10.1002/ece3.9342

**Published:** 2022-10-01

**Authors:** Ya Wang, Xijie Zhou, Jiajie Chen, Bin Xie, Lingfeng Huang

**Affiliations:** ^1^ Key Laboratory of the Ministry of Education for Coastal and Wetland Ecosystems College of the Environment and Ecology, Xiamen University Xiamen China; ^2^ Fujian Provincial Key Laboratory of Coastal Ecology and Environmental Studies Xiamen University Xiamen China; ^3^ Scientific Observing and Experimental Station of Fisheries Resources and Environment of East China Sea and Yangtze Estuary Ministry of Agriculture; East China Sea Fisheries Research Institute，Chinese Academy of Fishery Sciences Shanghai China

**Keywords:** climate change, East China Sea, HSI model, *Larimichthys crocea*, length‐based analysis, overfishing

## Abstract

Intense fishing pressure and climate change are major threats to the fish population and coastal fisheries. *Larimichthys crocea* (large yellow croaker) is a long‐lived fish, which performs seasonal migrations from its spawning and nursery grounds along the coast of the East China Sea (ECS) to overwintering grounds offshore. This study used length‐based analysis and habitat suitability index (HSI) model to evaluate the current life‐history parameters and overwintering habitat suitability of *L. crocea*, respectively. We compared recent (2019) and historical (1971–1982) life‐history parameters and overwintering HSI to analyze the fishing pressure and climate change effects on the overall population and overwintering phase of *L. crocea*. The length‐based analysis indicated serious overfishing of *L. crocea*, characterized by reduced catch, size truncation, constrained distribution, and advanced maturation causing a recruitment bottleneck. The overwintering HSI modeling results indicated that climate change has led to decreased sea surface temperature during *L. crocea* overwintering phase over the last half‐century, which in turn led to area decrease and an offshore‐oriented shifting of optimal overwintering habitat of *L. crocea*. The fishing‐caused size truncation may have constrained the migratory ability, and distribution of *L. crocea* subsequently led to the mismatch of the optimal overwintering habitat against climate change background, namely habitat bottleneck. Hence, while heavy fishing was the major cause of *L. crocea* collapse, climate‐induced overwintering habitat suitability may have intensified the fishery collapse of *L. crocea* population. It is important for management to consider both overfishing and climate change issues when developing stock enhancement activities and policy regulations, particularly for migratory long‐lived fish that share a similar life history to *L. crocea*. Combined with China's current restocking and stock enhancement initiatives, we propose recommendations for the future restocking of *L. crocea* in China.

## Graphical abstract

Length‐based analysis indicated serious overfishing of *Larimichthys crocea*, characterized by reduced catch, size truncation, constrained distribution, and advanced maturation. The overwintering HSI modeling results indicated that climate change has led to decreased sea surface temperature during *L. crocea* overwintering phase, which in turn led to area decrease and an offshore‐oriented shifting of optimal overwintering habitat of *L. crocea*. Hence, heavily fishing was the major cause of *L. crocea* collapse, while climate‐induced overwintering habitat suitability may have intensified the fishery collapse of *L. crocea* population

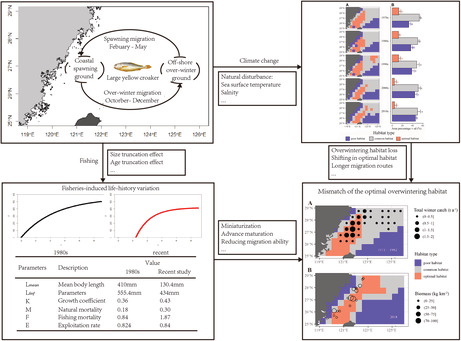

## 1 INTRODUCTION

Globally, heavily fishing activities and climate change are rapidly reducing the abundance of many marine organisms and increasing the likelihood of species extinction (Burgess et al., 2013; Cinner et al., 2012; Hoegh‐Guldberg & Bruno, 2010; Payne et al., 2016). For instance, intensive fishing and climate change have caused overfishing and declined catches in Canada, Iceland, and China (Du et al., 2014; Liang & Pauly, 2017; Pauly et al., 2001). Previous studies showed that fishing pressures and climate change can affect (i) the life‐history strategy of individuals, via impacts on physiology, morphology, and behavior (Ba et al., 2016; Olafsdottir et al., 2016); (ii) the population dynamics, via changes to key population processes throughout an organism's life history and habitat suitability (Perry et al., 2005). Hence, bottlenecks of any life‐history stage (e.g., spawning, hatching, larval survival, recruitment settlement, growth, and adult survival) and habitat suitability can cause overfishing of exploited species. In this context, recruitment bottleneck and habitat bottleneck are most well documented (Almany & Webster, 2006; Caddy, 2011). Correspondingly, the potential cause of overfishing is mismanagement because of a poor understanding of recruitment bottleneck and habitat bottleneck that constrain the productivity of the overall population.

Fishing alters the size structure by removing large fish exacerbated by size‐selective gear. Heavy fishing can diminish the ability of fish to reproduce (recruitment overfishing) and/or constrain the overall recruitment ability before they can fully realize their growth potential (growth overfishing) (Diekert, 2012) via size truncation effect (STE) (Berkeley et al., 2004; Froese et al., 2008; Langangen et al., 2019; Ottersen et al., 2006). This effect states that population shifts with decreasing body sizes and advancing maturation characteristic of the life‐history changes induced by fishing (Anderson et al., 2008; Bell et al., 2015; Berkeley et al., 2004). Hence, fishing for juveniles and mega‐spawner can weaken the reproductive potential of fish stock, called “recruitment bottleneck” (Doherty et al., 2004). Such bottlenecks are visible in long‐term time series and are a common cause of the collapse in intensely fished stocks, for example, in Western cod, Pacific rockfish, and North Sea ground fish (Froese et al., 2008; Harvey et al., 2006; Poulsen et al., 2007).

Climate change‐caused environmental conditions shift can have negative effects on the fish population (Graham et al., 2011; Johnson et al., 2011). In general, species' distribution patterns are relative to both life‐history strategies (Anderson et al., 2013) and physiology tolerance on environmental variables, such as sea surface temperature (SST), chlorophyll‐*a* concentration (Chl‐*a*), sea surface salinity (SSS), and currents (Guan et al., 2013; Yu & Chen, 2018). The environmental shift can selectively affect the habitat suitability of target species (Farrell et al., 2008). Lower habitat suitability of any life‐history stage can lead to species‐specific “habitat bottleneck” and later can have large consequences for loss of several fish's climatically suitable habitat, for example, Norwegian herring, Maine cod, and Mid‐Atlantic Bight winter flounder (Bell et al., 2015; Pershing et al., 2015).

Heavy fishing activities and shifts in environmental conditions can have combined effects on fishery collapse, especially for long‐lived species (Gascuel et al., 2014; Hsieh et al., 2009; Rose, 2004). Specifically, some studies suggested that long‐lived species are expected to have a slower demographic response to climate change (Berteaux et al., 2004; Wilson et al., 2010). Additionally, fishing‐caused STE can exacerbate long‐lived fish degradation via diminishing “bet‐hedging” capacity, including the ability to migrate and avoid poor areas, having flexibility in spawning times and locations, and production of high‐quality offspring that survive in a broader suite of environmental conditions, for adapting to rapid climate change (Bell et al., 2015). However, no example exists that demonstrates the STE and the climate‐induced effects on long‐lived migratory fish in the most heavily fishing (and minimally managed) marine ecosystem in the world: the East China Sea (ECS) (Szuwalski et al., 2017). To fill the knowledge gaps, we require a species that: first, under intensive fishing pressure; second, has specific habitat requirements; third, the habitat of which is affected by rapid climate‐induced habitat suitability variation; and fourth, has been reliably assessed over a long period by field surveys.

**FIGURE 1:**
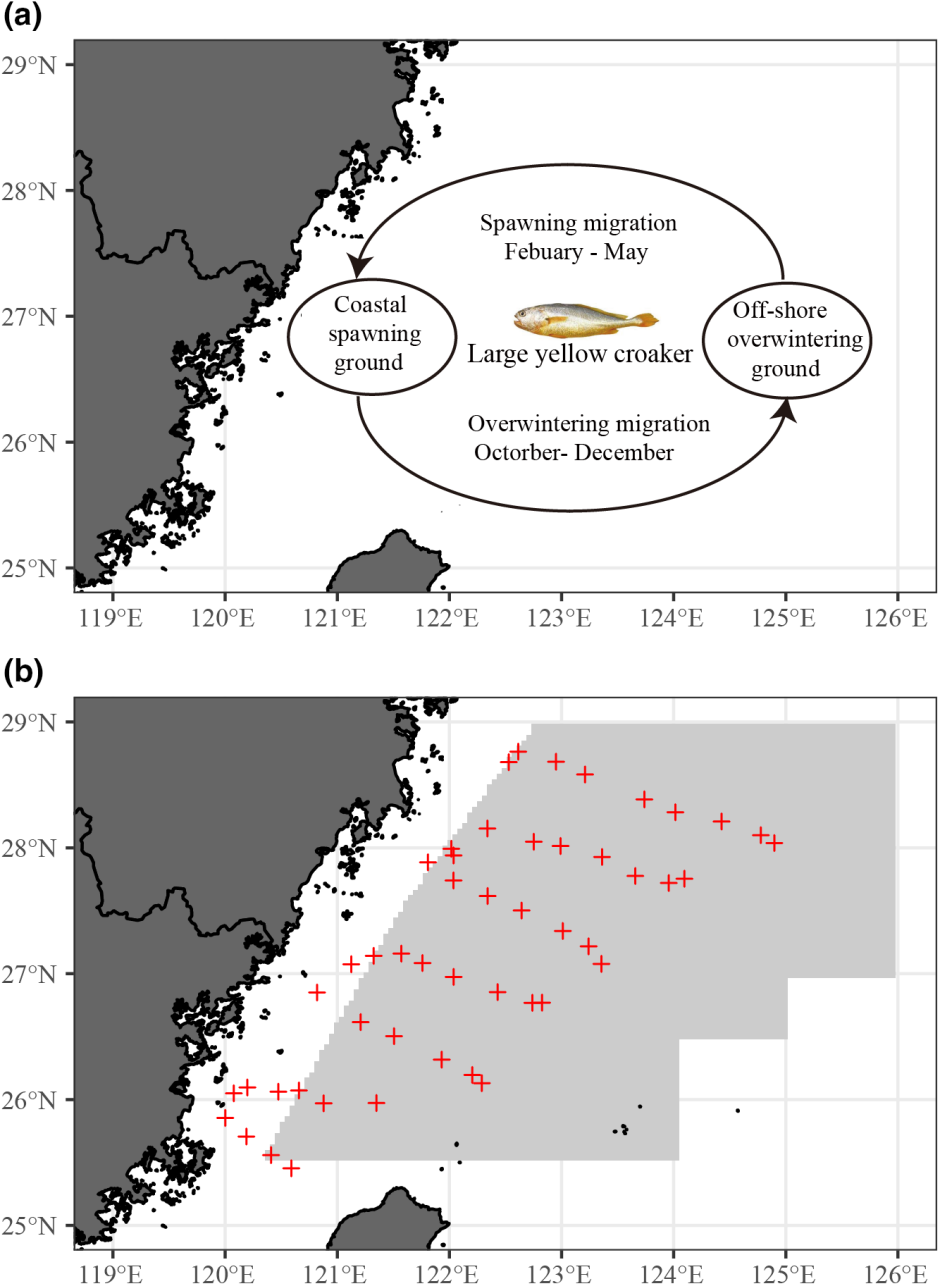
(a) Life‐history migration patterns of *Larimichthys crocea* in the ECS, e.g., *L. crocea* spawns inshore and “overwinters” offshore; and (b) the fishing area (gray area) during winter 1971–1982 and the survey stations (crosses) in the winter of 2018 for *L. crocea* in the mid‐southern East China Sea.

In the following, we provide an appropriate example by discussing changes in the specific population dynamic of an overexploited, long‐lived, migratory fish in the ECS, the large yellow croaker (*Larimichthys crocea*). The collapse of *L. crocea* represents an interesting example to explore both heavy fishing and climate change on the overall population: first, *L. crocea* ranked top among the four major marine economical fishes in China in the last century (Zhang et al., 2010) but suffered collapse since the 1980s. The latest International Union for Conservation of Nature (IUCN) Red List of Threatened Species labeled *L. crocea* as “critically endangered (CR)” (Liu et al., 2020). Second, *L. crocea* is a long‐lived species with maximum age of 21 years in the 1960s (Zhang et al., 2017). Accompanied by population collapse, the *L. crocea* population in the ECS is characterized by decreased maximum age and body size, and advanced maturation (Ye et al., 2012). Third, *L. crocea* is a migratory fish that conduct climatic migrations (e.g., movements driven by physiological tolerances of individuals to environmental factors such as temperature or salinity) and gametic migrations (e.g. movements that increase the reproductive success of individuals by promoting gonad development, increasing sexual encounter rates, or increasing the survival of offspring) between offshore water and coastal water during autumn–winter and spring–summer, respectively (Figure 1a).

## 2 MATERIALS AND METHODS

In this study, we only considered *L. crocea* in the mid‐southern ECS (120°E to 126°E, 25°N to 29°N) that have available data over long‐term series. Also, we evaluated only overwintering distribution patterns and overwintering habitat suitability because they are strongly linked to the physiology fitness and survival rate during the juvenile and adult stages and corresponding climatic migration phase of *L. crocea*.

### 2.1 Fishery data

To investigate how *L. crocea* population declines in the ECS in the last five decades, we analyzed two datasets on *L. crocea* in the ECS.

First, we used information from official commercial landing and hatchery release data. Catch data of *L. crocea* were collected from the *China Fishery Statistical Yearbook*. Annual catches during the year 1950–2019 in the ECS were calculated by adding the catch landing in the Jiangsu, Shanghai, Zhejiang, and Fujian provinces (Figure 1a). Hatchery release data in the ECS were obtained from Ningde Oceanic and Fishery Bureau by adding the number of official *L. crocea* hatchery release data in the Zhejiang and Fujian provinces (Figure 1a).

Second, to investigate how the geographical distribution of *L. crocea* shifts in the ECS, we analyzed a second dataset that includes both commercial fishery and scientific cruise data in the *L. crocea*'s overwintering ground (Figure 1b). Historical overwintering catches information (1970–1982) of *L. crocea* was obtained from the East China Sea Fisheries Research Institute. The dataset contains location and date, as well as the amount (in tons) of annual winter total catch (with 0.5° spatial resolution). Overwintering catch information and life‐history parameters (including length and maturation) for *L. crocea* were obtained from scientific cruise data in the period 2018–2019 through a bottom trawl survey in the mid‐southern ECS.

### 2.2 Environmental data

Three environmental factors were collected for *L. crocea* overwintering habitat suitability modeling: depth (m), sea surface temperature (°C), and sea surface salinity. The Bathymetry data (30 arc‐second spatial resolution) was obtained from Gridded Bathymetry Chart of the Oceans (GEBCO) (https://www.gebco.net/data_and_products/gridded_bathymetry_data/, accessed on September 2020) to represent depth. Due to data availability, monthly SST data in our study can only be obtained from two sources as follows: historical monthly SST and SSS data (0.5° spatial resolution) between 1971 and 2001 were acquired from the Simple Ocean Data Assimilation (SODA) reanalysis dataset (Carton & Giese, 2008) (from the Climate Data Library: http://iridl.ldeo.columbia.edu/, accessed on September 2020). Monthly SST data (4 km spatial resolution) between 2002 and 2019 were downloaded from the Moderate Resolution Imaging Spectroradiometer (MODIS) on board the satellite Aqua platform provided by the Ocean Biology Processing Group at NASA Goddard Space Flight Center (https://oceandata.sci.gsfc.nasa.gov/MODIS‐Aqua/Mapped/, accessed on September 2020). To investigate the environmental data from where fishing occurred, environmental data were resampled by the mean value of each month to 0.5° spatial resolution, then matched with the fisheries data.

### 2.3 Life‐history parameters of *L. crocea* in the ECS

To understand the life‐history parameters of *L. crocea* in the study area, we analyzed length frequency data with the electronic length frequency analysis (ELEFAN) approach using the *Tropfishr* package (Mildenberger et al., 2017). Size frequency of the commercial fishing catch during 1970–1982 is not available but we gathered the life‐history information from published literature (Liu & de Mitcheson, 2008; Xu et al., 1984a, 1984b; Ye et al., 2012; Yu & Lin, 1980) coinciding in time and space with the available data. We evaluated *L. crocea* life‐history parameters during 2018–2019 using body length data collected by otter trawl cruises, seasonally from November 2018 to November 2019 (e.g., November 18, 2018; January 20, 2019; April 18, 2019; July 17, 2019; September 28, 2019; November 18, 2019). We selected a total of 2074 *L. crocea* individuals between 2018 and 2019: the length (*n* = 2074) and weight (*n* = 853) of the collected *L. crocea* individuals were measured, and length–weight relationship was estimated based on the equation *w* = *aL* ^*b*^ (*n* = 853). The length frequency was calculated for each season for electronic length frequency analysis (ELEFAN) in *Tropfishr* package.

We fit the von Bertalanffy growth function (VBGF) through the length frequency and life‐history data to estimate life‐history parameters (e.g., maximum length, weight at length, length at maturity (*L*_mat_), and VGBF parameters including von Bertalanffy growth constant (*K*)) and asymptotic length (*L*_inf_), age at zero length (*t*_0_) (Brey & Pauly, 1986; Pauly & David, 1981; Sparre & Venema, 1998), and estimated mortality parameters (e.g., total mortality (*Z*), natural mortality (*M*), and fishing mortality (*F*)) (see details in Supporting Information). Specifically, the VBGF and mortality parameters were estimated following four approaches, including: (i) K‐Scan for the estimation of *K* for a fixed value of *L*_inf_ (ELEFAN K.S.); (ii) ELEFAN response surface analysis (ELEFAN R.S.A); (iii) ELEFAN with simulated annealing (ELEFAN S.A.); and (iv) ELEFAN with a genetic algorithm (ELEFAN G.A.). Among the above four workflows, ELEFAN R.S.A, ELEFAN S.A, and ELEFAN G.A. allow the simultaneous estimation of *K* and *L*_inf_ (Taylor & Mildenberger, 2017), while an advantage of ELEFAN S.A. and ELEFAN G.A. is the possibility to estimate all parameters of the seasonalized VBGF simultaneous (Scrucca, 2013; Xiang et al., 2013). Therefore, we estimated life‐history parameters using the 60 scenarios with different bins of length (bin = 10 and bin = 20), move average (MA) value (MA = 5, MA = 7, MA = 9, and MA = 11), and four different workflows (ELEFAN K.S., ELEFAN R.S.A., ELEFAN S.A., and ELEFAN G.A.) following Mildenberger et al. (2017).

For model validation and selection, we used all subsets model selection based on the fraction of the estimated sum of peaks (*R*_*n*_) following Gayanilo et al. (1997). Additionally, other ratios of life‐history parameters, such as *M*/*K* and *F*/*M*, were calculated with the estimated parameters. Moreover, Caddy et al. (1998) pointed out that the trophic level of a certain fish would be changing with size. Hence, we used the size and trophic levels relationship to estimate the size truncation effect on the overall population trophic level between the 1980s and a recent study (Supporting Information).

### 2.4 *L. crocea* overwintering distribution patterns and overwintering habitat suitability in the ECS

*L. crocea* is overall a habitat specialist during overwintering phase. Previous studies have shown that *L. crocea* has strong depth, temperature, and salinity preferences, while pH, dissolved oxygen, light intensity, sound, water velocity, and other factors may affect its distribution pattern, survival, and growth at different life stages (Liu, 2013; Wang et al., 2016, 2017). Unfortunately, detailed data on seasonal environmental data were limited between 1971 and 1982. Hence, only depth, SST, and SSS data were available as model inputs to determine suitable habitats for *L. crocea*. We used the HSI model to predict the overwintering habitat suitability of *L. crocea* in the ECS, which is a type of species distribution model (SDM) used for evaluating organisms–habitat relationships based on limited data or expert knowledge. In our HSI model, we used standardized abundance, HSI, as the response variable, and three environmental variables with the strongest correlation and the best data availability, depth, SST, and SSS as predictors. Firstly, we constructed both fitting‐based (Hua et al., 2020; Lee et al., 2019; Yu et al., 2020) and regression‐based (Chang et al., 2018; Jin et al., 2020) suitability index (SI) models to describe the relationship between each environmental variable and *L. crocea* abundance (Supporting Information). Then, we combined two types of SI models into HSI models, respectively. For each type of SI model, we used two empirical HSI models: the arithmetic mean model and the geometric mean model (Figure S1), under different environmental variable combinations (Lee et al., 2019).

For model validation and selection, we used catch data (abundance) from 1971 to 1980 and corresponding environmental data as training dataset, catch data from 1981 to 1982, and corresponding environmental data as testing dataset. We assumed a positive linear relationship between predicted HSI values and *L. crocea* abundance and evaluated the goodness of fit of the above relationship for each HSI model based on $R^{2}$ and the Akaike information criterion value adjusted for small sample size (AIC_c_) (Chang et al., 2013). A fitting‐based arithmetic mean model with two variables (e.g., depth and SST) yielded the maximum $R^{2}$ and the minimum AIC_c_ value (Supporting Information), thus was selected as the final HSI model. Correspondingly, fitting‐based SI model was selected as the best SI model. Finally, we retrained the SI model (see parameters and statistical test results in Supporting Information) and the HSI model using catch data (abundance) from 1971 to 1982 and corresponding environmental data.

We drew SI curves using the retrained final SI models. We then computed winter (December, January, and February next year) mean SST and used them to predict yearly winter mean HSI distribution between 1971 and 2019 with the retrained final HSI model, then calculated decadal winter mean HSI of the 1970s (1971–1980), 1980s (1981–1990), 1990s (1991–2000), 2000s (2001–2010), and 2010s (2011–2019). Regions with HSI value >0.7, 0.7 > HSI value >0.3, and HSI value <0.3 were regarded as optimal, average, and poor habitats, respectively. We computed the area of different habitat types each year and analyzed if there are significant differences in area between decades using Person one‐way ANOVA and Scheffe test.

## 3 RESULTS

### 3.1 Catch, hatchery release, and life‐history parameters of *L. crocea* in the mid‐southern ECS

**FIGURE 2:**
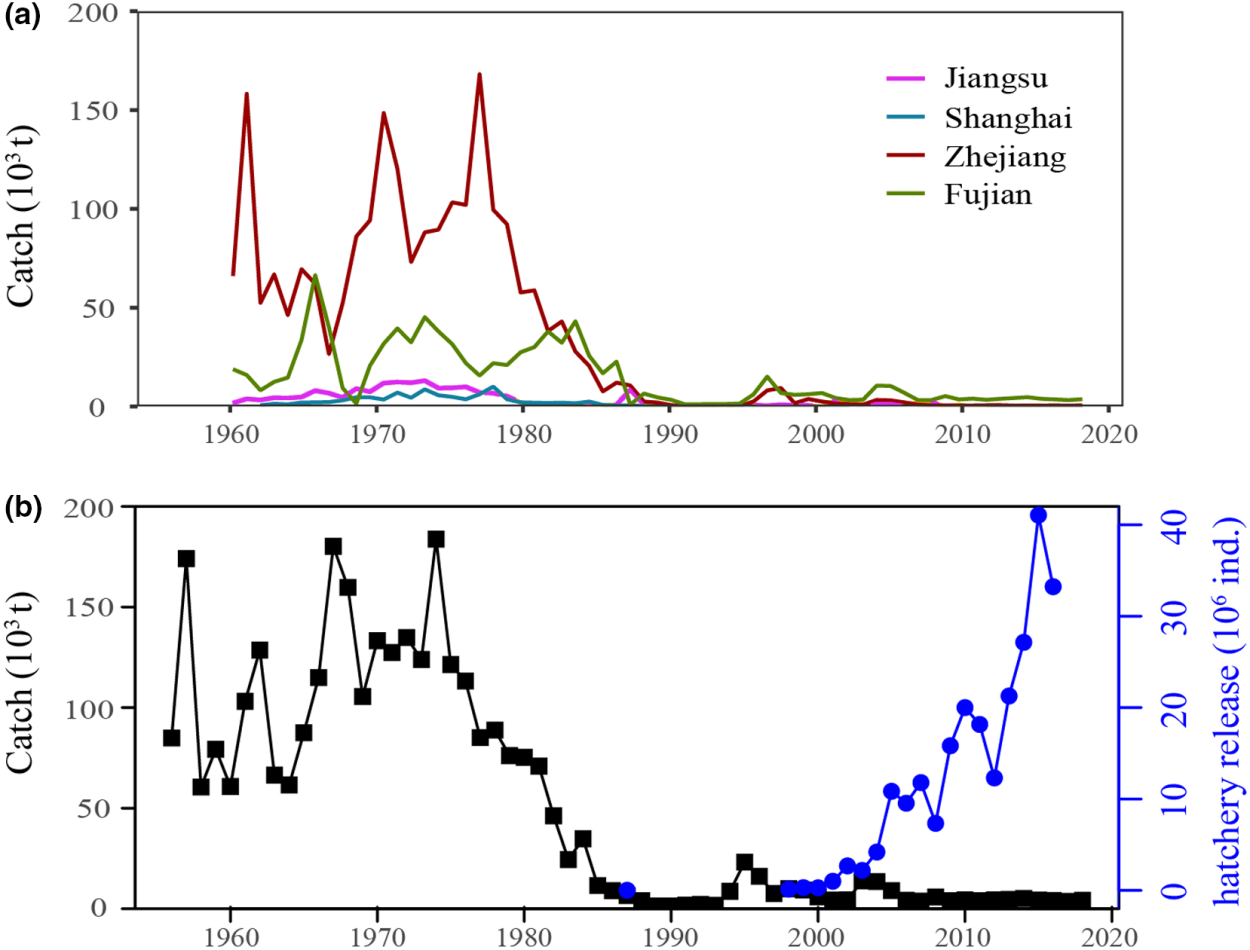
(a) Annual catch (1956–2019, 1000 tons) of *Larimichthys crocea* in Jiangsu, Shanghai, Zhejiang, and Fujian provinces, China; (b) total catch (1956–2019, 1000 tons) and the number of hatchery release of *L. crocea* at coastal Zhejiang and Fujian provinces (main catch and stock enhancement provinces in China).

Based on the reported landing information, the overall production of *L. crocea* in the ECS has been continuously declining since the 1970s. The variation in landing data indicated that the recent annual catch is now less than 4400 tons, which has declined by >90%, compared with peak yields of the 1970s (Figure 2a). Meanwhile, several long‐term and large‐scale restocking programs have been conducted since the 1990s. Millions of *L. crocea* have been released in the coastal areas of Fujian and Zhejiang provinces by the government (Figure 2b).

**FIGURE 3:**
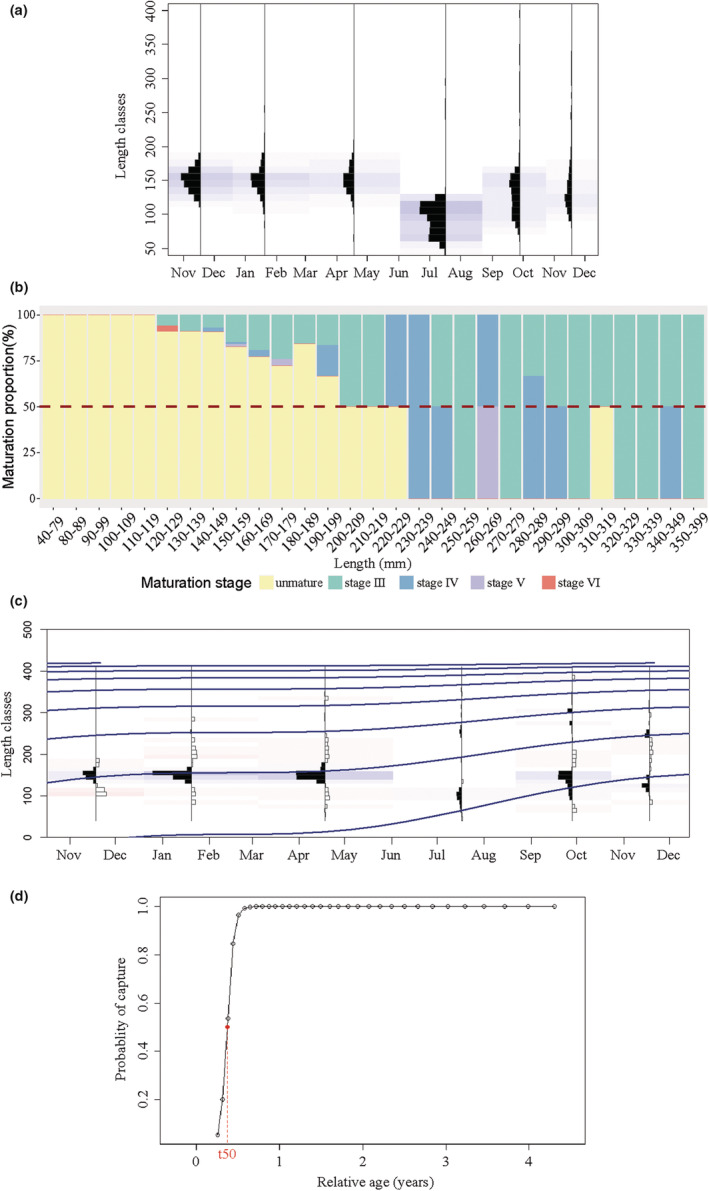
The graphical fit of current life‐history parameters and grow curves of *Larimichthys crocea* through length frequency data with electronic length frequency analysis (ELEFAN) of bin = 10 mm and moving average (MA) = 11 scenarios. (a) Histogram of length frequency distribution (bin = 10 mm); (b) histogram of maturation of *L. crocea*; (c) growth curve of *L. crocea* with ELEFAN S.A. (bin = 10 mm, MA = 11) scenario; and (d) graphical fit of catch probability of bin = 10 mm, MA = 11 scenario, and t_50_ represent the relative age of the first capture.

The life‐history parameter results show that since the 1980s, using length‐based data, we fit a series of ELEFAN models and most of the workflows were within feasible ranges for data‐limited measurement (Hordyk et al., 2015). The best model (ELEFAN S.A. with bin = 10, MA = 11, see details in Table S3) exhibited serious overfished status of *L. crocea* stock in the ECS (Table 1, Figure 3c). The most recent assessment found that (i) the average body length was 130.4 mm, with the body length of the predominant group being 145–155 mm (Figure 3a), while the average body weight was 34.5 g, with the dominant group weight being 10–50 g; (ii) the growth curve (Figure 3c) and maturation proportion (Figure 3b) show that juvenile *L. crocea* and the recruitment population have been the main catch targets, for which the age of first capture and age of probability 95% of capture are only 0.37 year and 0.49 year, respectively (Figure 3d); and (iii) the exploitation rate (*E*) of stock is now as high as 0.84, which reveals serious overfishing of *L. crocea* stock. Our results are consistent with previous studies that identified species as being smaller, younger, and maturing faster in ECS due to overfishing and STE. Firstly, both size truncation and age truncation have occurred in *L. crocea* population alongside with decrease of $L_{\inf}$ (555.4 mm in 1980s vs. 434 mm in 2018–2019), trophic level (∆TL = 0.15), the maximum age (8 years in 1980s vs. 6 years inLee et al., 2019), and predominant body length (144–155 mm). Also, the increase in body growth rate from the 1980s to the current study (*K*: 0.36 vs. 0.43, respectively) is consistent with advanced and smaller size at maturity (*L*_mat_: 350–400 mm vs. *L*_mat_: 200–205 mm, respectively) (Table 1). Still, the fishing mortality (*F*) and exploitation rate (*E = F/Z*) were predicted to be 1.57 and 0.84, respectively, which are continuously increasing compared with the 1980s (Table 1).

**TABLE 1:** Current life‐history parameters fitted with ELEFAN S.A. of bin = 10 mm, MA = 11 scenario, and historical life‐history parameters of *Larimichthys crocea*.

Parameter	Description	Current (2019)	History (1980s)
Life‐history			
*a*, *b*	Length–weight relationship	2.60 × 10^−6^ (mm, g) 3.26	
*L*_mean_	Mean body length	130.4 mm	410 mm
*L*_inf_	Asymptotic length	434 mm	555.4 mm
*L*_mat_	Size of maturity	200–210 mm	350–400 mm
*K*	Growth coefficient	0.43	0.36
*M*	Natural mortality	0.30	0.18
*F*	Fishing mortality	1.57	0.84
*Z*	Total mortality	1.87	1.02
*E*	Exploitation rate	0.84	0.824
*t*_0_	Age of length zero	−0.06 year	
*t*_50_	Age of first catch	0.37 year	
*t*_95_	Age of probability 95% of capture	0.49 year	
Goodness of fit			
*R*_*n*_	The score value of true parameter can be calculated by the model	0.61	

### 3.2 Overwintering habitat suitability of *L. crocea* in the ECS

We used both fitting‐based and regression‐based methods to construct SI models of each environmental variable and employed both the arithmetic mean model and geometric mean model under different environmental variable combinations to calculate HSI values. A fitting‐based arithmetic mean model with two variables (e.g., depth and SST) yielded the maximum $R^{2}$ and the minimum AIC_c_ value (Supporting Information), thus was selected as the final HSI model. The statistical analysis of fitting‐based SI models (Supporting Information) shows they were all significant (*p* < .05). As shown by the SI curves (Figure S1), the optimal range for depth, SST, and SSS during winter in our study area was 36–72 m, 18.2–20.5°C, and 33.89–34.27, respectively.

**FIGURE 4:**
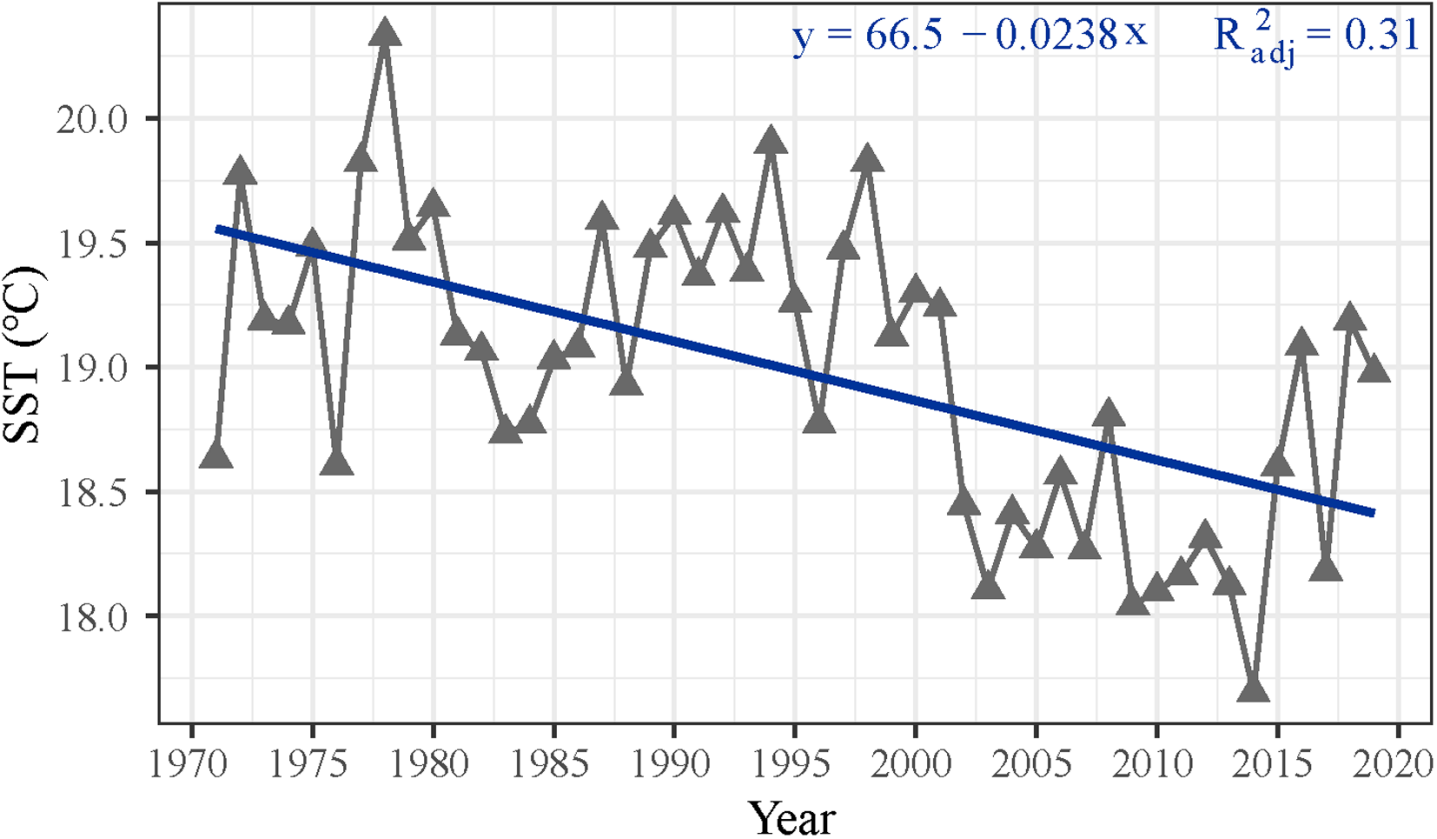
Annual winter season sea surface temperature (SST) anomalies (gray triangles) from 1970 to 2019. Showing long‐term SST decline from 1970 to 2019 (blue solid line).

**FIGURE 5:**
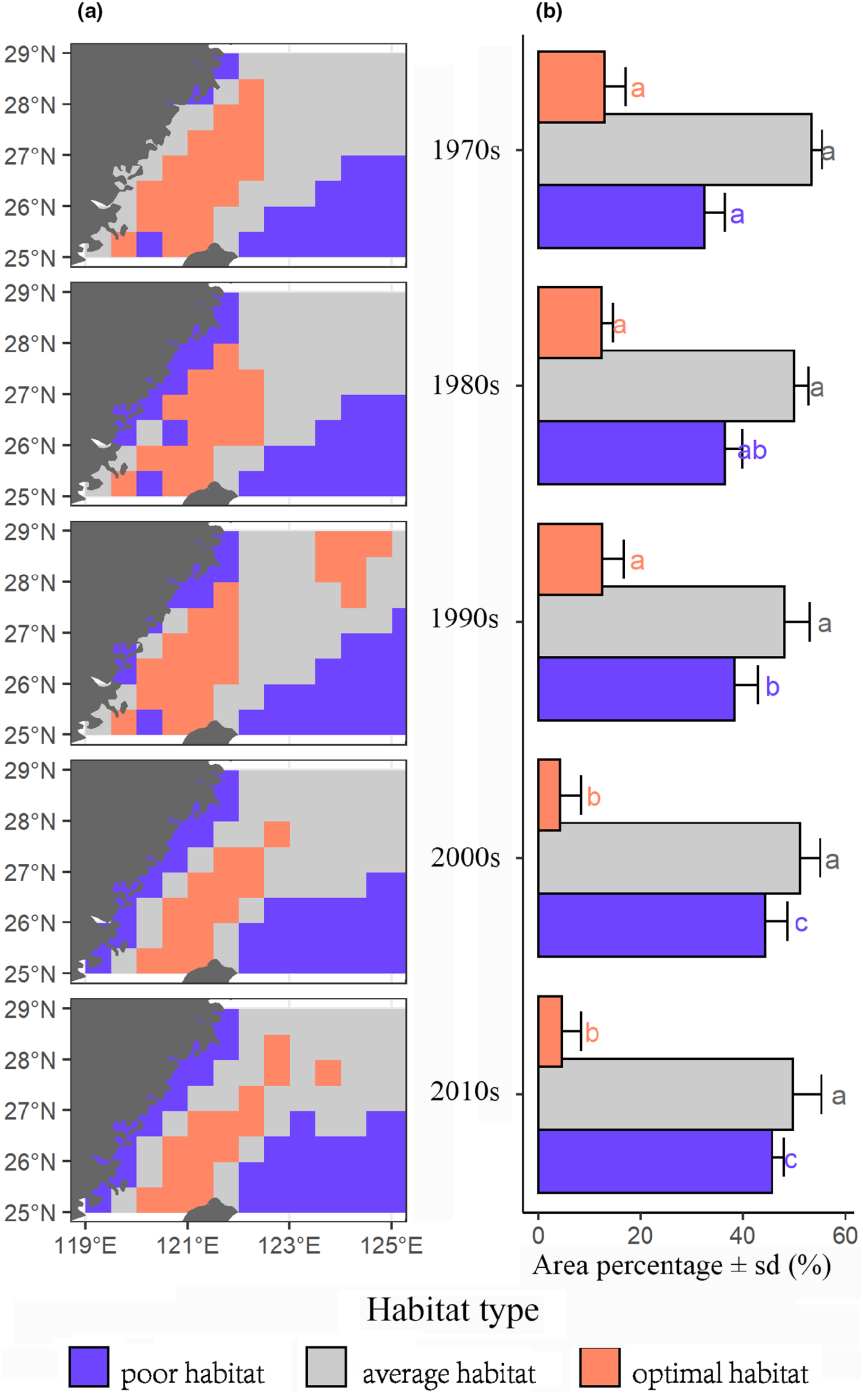
Decadal variations in (a) spatial distribution of predicted habitat suitability and (b) area percentage of optimal, average, and poor habitat since the 1970s. The areas with habitat suitability index (HSI) value >0.7, 0.7 > HSI value >0.3, and HSI value <0.3 were regarded as optimal, average, and poor habitat, respectively.

The recent five decades' cooling trend in winter is remarkable, with the reduced average SST (−0.028°C/year, *R* ^2^ = .31, *p* < .05) between 1982 and 2019 in the mid‐southern ECS in winter (Figure 4). The cooling trend in our study area may be influenced by the Kurushio extension; specifically, in the latest IPCC report (2019), the Kurushio extension exhibits long‐term cooling, which is consistent with our result. Also, another study revealed the cooling trend along China's and Japan's coast (−0.69 ± 0.44°C/decade), opposing the overarching global warming trend, especially in the winter season due to the extreme cold events (Bindoff et al., 2019; Liao et al., 2015). Consisted with the cooling trend of SST during the overwintering phase of *L crocea*, the results of HSI models show the mean habitat overwintering suitability of the 1970s (1971–1980), 1980s (1981–1990), 1990s (1991–2000), 2000s (2001–2010), and 2010s (2011–2019) shifting in our study area. Figure 5a shows that there was no significant change (*p* > .05) in the average and optimal habitat area from the 1970s to 1990s. However, the percentage of optimal habitat decreased significantly (*p* < .05) from 13%, 12%, and 13% in the 1970s, 1980s, and 1990s to 4% and 5% in the 2000s and 2010s. Figure 5b shows that the spatial distribution of habitat suitability also changed: the optimal area has moved toward a southeast direction, with suitable habitats becoming offshore oriented. Unfortunately, regarding data availability, the HSI models conducted in our study may be biased because we used catch data during 1971–1982 as a measure of abundance (e.g., highly dependent on the effort). Hence, abundance data obtained from the scientific cruise is more convincing than using catch data as abundance data and should be encouraged in future studies.

## 4 DISCUSSION

### 4.1 Fishing‐induced life‐history variation

Overall, this study provides evidence of serious fishing‐induced life‐history variation in *L. crocea* population and represents a glimpse of fishery collapse. The observed life‐history parameters show that the body size of *L. crocea* has on average, decreased during the last five decades (Table 1). Previous studies in the 1970s and 1980s revealed that the main catch of *L. crocea* consisted of 2‐ or 3‐year‐old (400–800 g) individuals (Yu & Lin, 1980), while 95% of catches in this study were individuals aged 0 or 1 year. Still, we indeed find that the maturation proportion of *L. crocea* is dramatically declined during the last five decades (Figure 2b). It is likely that the decrease in average body size, size truncation, and maturation proportion is affected by continuous fishing pressures and indiscriminate fishing (~50% trawl with 54 mm mesh size) in China (Gaichas et al., 2014; Kirby, 2004; Kuparinen et al., 2016). Previous studies have identified continuous fishing pressures can erode fish biomass by substantially decreasing the proportion of large individuals, and subsequent fishing‐induced life‐history variation is likely to have negative effects on overall population structure and recruitment (Johnston & Temple, 2002; McMahan et al., 2020; Morita et al., 2005). For instance, the intense commercial fishing in Australia has caused a recruitment bottleneck, even the extinction of some populations, such as the collapse of Eastern blue groper, gemfish, and blue‐eye trevalla (Last et al., 2011).

Fishing‐induced life‐history variation may also constrain species distribution because the migratory ability of a species is strongly dependent on dispersal characteristics, such as morphological traits (Hsieh et al., 2009). To substantiate our finding on the potential negative effect of life‐history variation on the overall population, we compared the historical and recent distribution of *L. crocea*. The result showed that ~70% of potential catch areas have disappeared (Figure S2), with the highest disappearance rate in offshore areas (122°E–125°E), which follows the life‐history variation in *L. crocea* during the last five decades. Consequently, constriction of geographic distribution, associated with a decline in body size, may reduce the ability to respond to climatic stress, by limiting movement (Reusch et al., 2005).

### 4.2 Climate‐induced overwintering habitat degradation may intensify the effect of overfishing

The best HSI model (next best model: ∆AIC_c_ = 2, for other models, Table S2) to explain catch patterns under unexploited status over time included SST and depth. The result of SI suggested that the optimal overwintering temperature range (18.2–20.5°C) and depth (36–72 m) of *L. crocea* mirror previous lab‐based observation of optimal growth temperature (17–24°C) and empirical observation of optimal overwintering depth (50–60 m) (Liu, 2013; Xu & Chen, 2011). It is worth noting that SST in the mid‐southern ECS has decreased by an average of 1°C between the 1980s and 2010s with an annual decrease in SST rate —0.028°C/year (Figure 4). HSI variation results in the last five decades suggest that the cooling trend of SST in the ECS has significantly reduced the proportion of optimal and average overwintering habitats for *L. crocea* (Figure 5b).

Consequently, in response to the SST decrease in winter, migratory species, like *L. crocea*, are expected to respond in two ways as follows. Generally, marine organisms respond to climate change through shifts in distribution (Guisan & Thuiller, 2005). For instance, in the North Sea, both exploited and unexploited fish species have shifted to higher latitudes and deeper water between 1977 and 2001 in response to rising sea temperatures (Perry et al., 2005); in the Eastern Tropical Pacific, demersal species were projected to move into shallow water by the mid‐21st century in response to high greenhouse gas emissions (Representative Concentration Pathways, RCP8.5) and strong migration (RCP2.6) scenarios (Clarke et al., 2020). Alternatively, it is also commonly observed that species stay in poor habitats against climate change but suffered climate‐induced life‐history variation. Particularly, it occurs when marine fishes are living in temperatures outside their physiology optima: this results in reduced aerobic scope, which negatively affects their growth and reproduction (Pearson & Dawson, 2003; Pörtner & Knust, 2007; Toresen et al., 2019). Hence, species like *L. crocea* must either migrate to remain within a suitable habitat or suffer the consequences (Bell et al., 2015). Interestingly, *L. crocea* overwintering distribution pattern did not shift alongside a decrease in winter SST, which is a good indicator that temperature per se did not explain the overall shift of *L. crocea* distribution. Such absence of a clear systematic impact of temperature may be due to the life‐history parameters degradation, which could constrain the hedging capacity against climate change (Thorson et al., 2017). For example, STE caused a change in the length age structure is the main diver of interannual shifts in summer flounder distribution, while temperature had little influence on the change in distribution (Bell et al., 2015).

**FIGURE 6:**
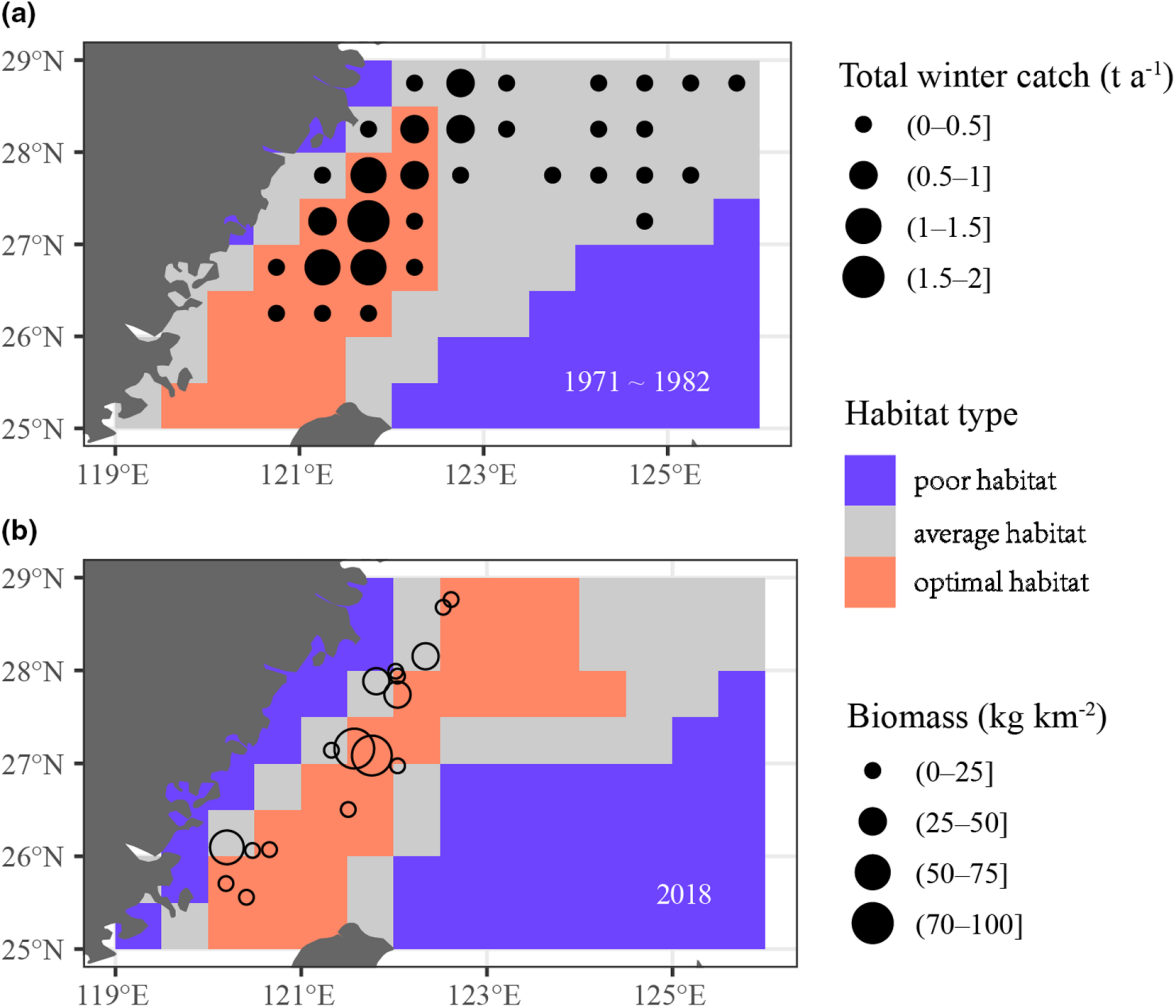
Spatial distribution of (a) mean winter total catch of *Larimichthys crocea*, overlaid with the predicted HSI map in 1971–1982 and (b) biomass of *L. crocea* overlaid with the predicted HSI map in 2018.

More broadly, the “match/mismatch hypotheses” may explain the combined effects of heavy fishing and climate change on the decrease in the overall population (Cushing, 1990; Edwards & Richardson, 2004). Based on our study, we suggest that fishing‐induced life‐history variation leads to the “mismatch” of *L. crocea* optimal overwintering habitat. Specifically, our study demonstrated that *L. crocea* has both life‐history variation and size truncation compared with the 1980s, with significantly smaller body size and advanced maturation (Figure 2b). This truncation in overall size structure can significantly affect swimming ability, such as reducing the sustained swimming time and average swimming speed, namely size dependent, consequently reducing the distribution range of *L. crocea* (Jorgensen et al., 2008; Opdal & Jørgensen, 2015, 2016). Given the climate‐induced changes in overwintering habitat suitability that occurs in the mid‐southern ECS, the fishing‐induced life‐history population variation that constrains dispersal capability could pose a significant “mismatch” of optimal overwintering habitat to *L. crocea*‐like migratory species (Figure 6). Such applicability of “mismatch hypotheses” to the specific long‐lived migratory fish exposed to fishing and climate change had rarely been demonstrated.

### 4.3 Management implication

In China, approximately 50% of China's fish stocks have been overexploited or collapsed (Cao et al., 2017). Our approach can provide insight in anticipation of stock enhancement and management that may facilitate conservation and re‐stocking. While our results highlight that, as well as the previously described “lack of timely, effective or sufficient management, combined with heavy fishing pressure, particularly at spawning and overwintering grounds were major factors responsible for croaker stock declines” (Liu & de Mitcheson, 2008), climate change‐induced overwintering habitat is another potential reason for the stock depletion. This is highly worrying because long‐lived migratory fish like *L. crocea* decline even faster were both heavy fishing and climate‐induced habitat suitability synergies (Färber et al., 2018).

The severe situation has led to an urgent need to re‐evaluate fishery management and calls for a species‐specific or life‐history‐based approach to stock enhancement (Dubik et al., 2019; Lotze et al., 2011; Pinsky et al., 2018; Young et al., 2006). First, regarding the fishing‐caused size truncation effects, the deficiencies in China's input control allow fishers to conduct indiscriminate intense fishing on large individuals of long‐lived species after seasonal closure and consequently, alter the dynamics of the harvested species and the ecosystem (Shen & Heino, 2014; Su et al., 2020). Hence, if capture fishery activities are not fully regulated scientifically and deliberately, restocking long‐lived migratory fish will be difficult. Here, we suggest establishing stricter input controls on the fishery, including reducing the fishing capacity and efforts, eliminating unregistered/illegal fishing vessels, increasing the minimum mesh‐size standard, and adopting output controls, especially for single‐species total allowable catch (TAC) or/and ecosystem TAC of *L. crocea*‐like species. Secondly, regarding the climate‐induced overwintering habitat, we recommend designing seasonal special reserve zones and more targeted regulations in crucial *L. crocea* habitats, which should be managed like MPAs. Ultimately, our application of HSI model illuminates the mechanisms of fishing‐induced life‐history variation and climate change‐caused “mismatch” impacts on long‐lived migratory species (Wilson et al., 2008). Also, fishery managers often deploy hatchery releases to address the recruitment bottleneck of species' restocking (Kitada, 2018; Myers et al., 2004; Taylor et al., 2017). Because *L. crocea*'s suitable overwintering habitats have shifted toward offshore areas, to tackle both recruitment and habitat bottleneck, we recommend that stakeholders choose larger juveniles, even mega‐spawner for hatchery release to keep pace with the shifting of suitable habitats caused by climate change.

## AUTHOR CONTRIBUTIONS

**Ya Wang:** Conceptualization (equal); data curation (equal); formal analysis (lead); funding acquisition (equal); investigation (lead); methodology (lead); project administration (equal); resources (equal); software (lead); supervision (supporting); validation (equal); visualization (lead); writing – original draft (lead); writing – review and editing (lead). **Xi Jie Zhou:** Conceptualization (lead); data curation (lead); formal analysis (lead); funding acquisition (supporting); investigation (equal); methodology (lead); project administration (supporting); resources (equal); software (lead); supervision (equal); validation (equal); visualization (lead); writing – original draft (lead); writing – review and editing (lead). **Bin Xie:** Conceptualization (supporting); data curation (supporting); formal analysis (supporting); funding acquisition (equal); investigation (lead); methodology (supporting); project administration (supporting); resources (equal); software (supporting); supervision (supporting); validation (supporting); visualization (supporting). **Jiajie Chen:** Conceptualization (equal); investigation (lead); project administration (equal); validation (equal); writing – review and editing (equal). **Lingfeng Huang:** Conceptualization (lead); funding acquisition (lead); methodology (equal); project administration (lead); writing – original draft (equal); writing – review and editing (lead).

## CONFLICT OF INTEREST

The authors declare no conflict of interest.

### OPEN RESEARCH BADGES

This article has earned Open Data and Open Materials badges. Data and materials are available at https://doi.org/10.5061/dryad.08kprr538.

## Supporting information


Appendix S1

## Data Availability

The data generated for this study are available at Dryad Data Repository: https://doi.org/10.5061/dryad.08kprr538.
