# Supplementary material for: Climate‐induced habitat suitability changes intensify fishing impacts on the life history of large yellow croaker (Larimichthys crocea)
Source: Ecol Evol. 2022 Oct 1;12(10):e9342. doi: 10.1002/ece3.9342 (PMC9526033; doi:10.1002/ece3.9342)
Supplement: Supplementary file 1 — Appendix S1 [file ECE3-12-e9342-s001.docx]

**Supporting information**

**Appendix 1 – Additional materials and methods information**

**Supplementary methods of *L. crocea* ELEFAN model**

The R package TropfishR (version 1.6.1) (Mildenberger et al. 2017), which integrates a set of classical stock assessment methods using length frequency (LFQ) data (Sparre and Venema 1998), was used to derive growth parameters of the von Bertalanffy growth function (VBGF) and seasonal oscillating VBGF (soVBGF) (Pauly 1990, Somers 1988). TropfishR includes routine methods to (1) calculate VBGF parameters; (2) estimate mortality parameters; (3) conduct virtual population analyses (VPA); (4) assess stock status and predict yield. In this study, the ELEFAN was used to estimate von Bertalanffy growth constant (*K*), asymptotic length (*L_inf_*), total mortality (*Z*), natural mortality (*M*) and fishing mortality (*F*). In TropFishR, there are four different methods, based on the ELEFAN functionality: (i) K–Scan for the estimation of *K* for a fixed value of *L_inf_*; (ELEFAN K.S.); (ii) ELEFAN Response Surface Analysis (ELEFAN R.S.A); (iii) ELEFAN with simulated annealing (ELEFAN S.A.); (iv) ELEFAN with a genetic algorithm (ELEFAN G.A.), where the last three methods allow the simultaneous estimation of K and *L_inf_*.

The growth was modelled according to VBGF and soVBGF as describe by Pauly and David (1981) and Somers (1988) as equation (1) and equation (2), respectively:

(1) $L_{t}=L_{inf}(1-\exp\left( -K\left( t-t_{0} \right) \right))$

(2) $L_{t}=L_{inf}(1-\exp\left( -K\left( t-t_{0} \right)+S(t \right)-S(t_{0})$))

Where *L_t_* is length at age *t*, *L_inf_* is asymptotic length, *K* is von Bertalanffy growth constant, *t_0_* is age at length 0.$S(t)=\left( {CK}/{2\pi} \right)\sin2\pi\left( t-t_{s} \right)$, where *C* is constant indicating the amplitude of the oscillation, *t_s_* is the fraction of a year where the sine wave oscillation begins, which reflect the time of year when growth turn positive and more biological meaningful.

The estimation methods of other life-history parameters, including rate the age at zero length and the total mortality, were identical for all ELEFANs in subsequent steps. The age at zero length *t_0_* was calculated with the empirical equation based on *L_ꝏ_* and K (Pauly 1983). The equation was given as:

(3) ${log}_{10}\left( -t_{0} \right)=-0.3922-0.275\times{log}_{10}\left( L_{inf} \right)-1.038\times{log}_{10}(K)$

The Total mortality rate Z was obtained by linearizing the catch curve to length–frequency data and modeled as Pauly (1990), which was given as:

(4) $\ln\left( N_{t} \right)=\ln\left( N_{0} \right)-Z_{t}$

We used growth-based method advocated by Then et al. (2015), which is based upon a meta-analysis of 201 fish species, to estimate the natural mortality (*M*). The power function was acquired by fitting Then’s model, which was given as:

(5)$M=4.118\times K^{-0.73}L_{inf}^{-0.33}$

We used the fraction of the estimated of peaks (*R_n_*) to evaluate the fitness of ELEFAN performance advocated by Gayanilo & Pauly (Gayanilo et al. 1997), which was given as:

(6) $R_{n}$=${10}^{(ESP/ASP)}/10$

Where the *ESP* is the ‘estimate sum of ‘peaks’ crossed by the estimated growth curves, the *ASP* is the ‘available sum of peaks’, which represent the maximum possible score and is calculated as the sum of the highest values in positive scored peaks, the *ESP/ASP* indicates the goodness of fit with ELEFAN (Pauly 1987) and the maximum possible for *R_n_* should be 1.

Maturation (or fecundity) analysis of *L.crocea* in 2018-2019 were conducted in laboratory, the classification of maturation stage (StageⅠ–Ⅵ) of *L.crocea* were identified based on China’s *Specifications for Oceanographic Survey–Part 6: Marine Biological Survey*. Where the stageⅠand stageⅡ were unmature stage, while stage Ⅲ–Ⅵ were identified as mature stage following Lin’s definition (Lin et al. 2004).

Caddy et al (1998) pointed out that the trophic level of a certain fish would be changing with size. Here, we used size and trophic levels relationship to estimate the size truncation effect on overall population trophic level between 1980s and recent study. Here, we used size and trophic levels relationship summarized as:

(7) $\Delta TL=b\times({log}_{10}L_{previous}-{log}_{10}L_{recent})$

Where *b* = 0.63 for higher-order carnivores (with adult TL higher than 3.75), *L_previous_* represents the body size of *L. crocea* in 1980s and *L_recent_* represents the recent body size of *L. crocea*.

**Supplementary methods of** ***L. crocea* overwintering HSI model**

The regression-based SI models was built through the following steps. Firstly, we conducted smooth spline regression for *L. crocea* abundance and each environmental factor to obtain the response curves of abundance to environmental factors. Secondly, the values of the response curves were converted/standardized into SI models using equation (8)

(8) $SI=\frac{Y-Y_{min}}{Y_{max}-Y_{min}}$

where Y is the predicted value of abundance, $Y_{min}$ and $Y_{max}$are the minimum and maximum values of the predicted abundance between 1971 and 1982, respectively.

To build the fitting-based SI models, we first calculated observed SI values. For each environmental variable, we divided the appearance range into equal class intervals, using the classInt package (Bivand et al. 2020). Observed SI value in the $i_{th}$ class interval was calculated using equation (9) (Chang et al. 2013).

(9) ${SI}_{i}=\frac{Y_{i}-Y_{min}}{Y_{max}-Y_{min}}$

Where $Y_{i}$ is the mean abundance in the$i^{th}$ interval; $Y_{min}$ and $Y_{max}$ are the minimum and maximum abundance in all class intervals, respectively. Then, we calculated parameters of SI models using observed SI values. The SI model of depth, SST and SSS were estimated using equation (10), (11) and (12) modified from Chen et al (Chen et al. 2009).

(10) ${SI}_{depth}=exp\left[ a\times\left( X_{depth}-b \right)^{2} \right]$

(11) ${SI}_{SST}=exp\left[ a\times\left( \left( X_{SST} \right)^{2}-b \right)^{2} \right]$

(12) ${SI}_{SSS}=exp\left[ a\times\left( \left( X_{SSS} \right)^{2}-b \right)^{2} \right]$

Where a and b are the estimated model parameters, which were solved with a least square estimate to minimize the residuals; $X_{depth}$, $X_{SST}$ and $X_{SSS}$ are the class interval values for each variable.

For both regression-based and fitting-based SI models, we assumed a linear relationship between abundance of *L. crocea* and predicted SI values and the goodness-of-fit of the above linear relationships for SI models was evaluated using F test and $R^{2}$. To test the year-to-year HSI variations would affect the comparison, we have added a year-to-year comparison between HSI (e.g. year-to year HSI estimation with all data from 1971-1980) and HSIs (e.g. year-to-year HSI estimation with data from 1971-1980 except that year). The comparison showed that the estimation results of HSI and HSIs are similar, which revealed the year-to-year variation is not likely to affect the HSI result significantly.

**Appendix 2 - Additional Figures and Tables**


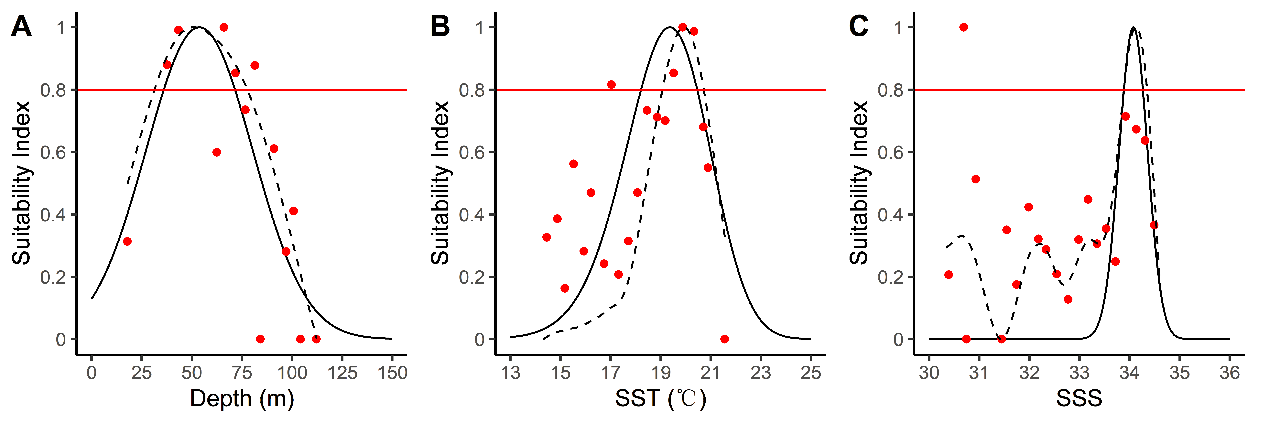


Figure S1. Suitability index curves of (A) depth; (B) sea surface temperature and (C) sea surface salinity of *L. crocea* using fitting-based methods (solid line) and regression-based methods (dashed line). The intersections of the suitability index curves and the red line (Suitability index = 0.8) denote the optimal range of each environmental variable. We finally used fitting-based SI models and curves in our results.


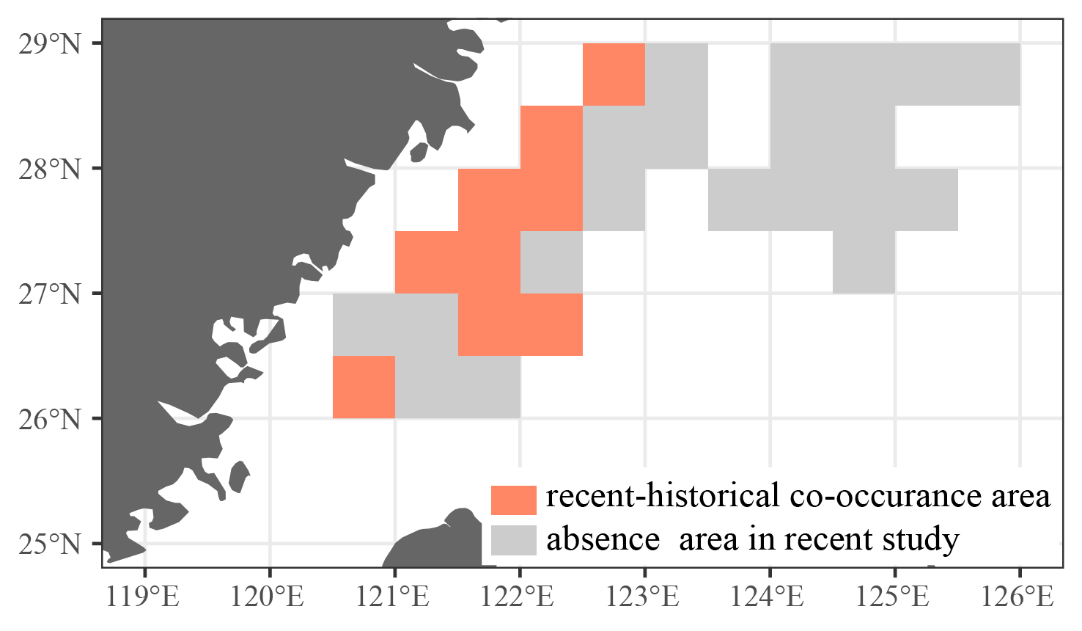


Figure S2. Comparison between the current and historical occurrence of *L. crocea*, contrasting areas of current (red) and historical (grey) occurrences.

Table S1 Parameters and statistical test of SI models using fitting-based methods (solid line) and regression-based methods (dashed line).

| Methods | SI model | a | b | df | F | P | $R^{2}$ (%) |  |
| --- | --- | --- | --- | --- | --- | --- | --- | --- |
| Fitting-based | $\mathrm{SI}_{depth}$ | -0.0007069 | 53.80 | - | 10.87 | <0.05 | 7.26 |  |
|  | $\mathrm{SI}_{SST}$ | -0.0001187 | 375.2 | - | 18.14 | <0.05 | 11.54 |  |
|  | $\mathrm{SI}_{SSS}$ | -0.001346 | 1161 | - | 31.70 | <0.05 | 18.57 |  |
| Regression-based | $\mathrm{SI}_{depth}$ | - | - | 3.51 | 13.44 | <0.05 | 8.41 |  |
|  | $\mathrm{SI}_{SST}$ | - | - | 5.09 | 29.01 | <0.05 | 16.43 |  |
|  | $\mathrm{SI}_{SSS}$ | - | - | 8.33 | 38.47 | <0.05 | 21.62 |  |

Table S2 Statistical test results of linear regression model between abundance of *L. crocea* and predicted HSI values and AIC (Akaike’s Information Criterion) value for HSI models. HSI models are based on fitting-based or regression-based SI models and using both the arithmetic mean model and the geometric mean model under different environmental variable combinations.

| HSI model | Fitting-based | | | Regression-based | | |
| --- | --- | --- | --- | --- | --- | --- |
|  | P(F) | $R^{2}$(%) | AIC | P(F) | $R^{2}(\%)$ | AIC |
| $HSI=\mathrm{SI}_{depth}$  ${HSI=SI}_{SST}$  $HSI=\mathrm{SI}_{SSS}$ | >0.05 | 5.65 | 237 | >0.05 | 8.416 | 94.39 |
|  | <0.05 | 19.01 | 232 | <0.05 | 12.32 | 92.73 |
|  | >0.05 | 7.97 | 236 | >0.05 | 8.61 | 94.31 |
| $HSI=\left( \mathrm{SI}_{depth}+\mathrm{SI}_{SST} \right)/2$  $HSI=\left( \mathrm{SI}_{depth}+\mathrm{SI}_{SSS} \right)/2$  $HSI=\left( \mathrm{SI}_{SST}+\mathrm{SI}_{SSS} \right)/2$  ${HSI=\left( \mathrm{SI}_{depth}+\mathrm{SI}_{SST}+\mathrm{SI}_{SSS} \right)}/2$ | <0.05 | 31.50 | 225 | <0.05 | 24.16 | 87.22 |
|  | <0.05 | 19.26 | 231 | <0.05 | 21.32 | 88.62 |
|  | <0.05 | 13.91 | 234 | <0.05 | 12.31 | 92.73 |
|  | <0.05 | 22.3 | 230 | <0.05 | 20.97 | 88.78 |
| $HSI=\sqrt{\mathrm{SI}_{depth}\times\mathrm{SI}_{SST}}$  $HSI=\sqrt{\mathrm{SI}_{depth}\times\mathrm{SI}_{SSS}}$  $HSI=\sqrt{\mathrm{SI}_{SST}\times\mathrm{SI}_{SSS}}$ | <0.05 | 28.98 | 227 | <0.05 | 22.93 | 87.83 |
|  | <0.05 | 16.51 | 233 | <0.05 | 15.82 | 91.18 |
|  | <0.05 | 13.40 | 234 | <0.05 | 13.27 | 92.32 |
|  | <0.05 | 18.84 | 232 | <0.05 | 18.70 | 89.86 |

Table S3 The year-to-year comparison between HSI (e.g. year-to year HSI estimation with all data from 1971-1980) and HSIs (e.g. year-to-year HSI estimation with data from 1971-1980 except that year).

| YEAR | HSI | HSIs |
| --- | --- | --- |
| 1971 winter | 0.366762 | 0.350818 |
| 1972 winter | 0.422388 | 0.4276 |
| 1973 winter | 0.414701 | 0.410946 |
| 1974 winter | 0.417538 | 0.433221 |
| 1975 winter | 0.435848 | 0.445246 |
| 1976 winter | 0.414817 | 0.417584 |
| 1977 winter | 0.445974 | 0.446364 |
| 1978 winter | 0.423181 | 0.401825 |
| 1979 winter | 0.464594 | 0.413585 |
| 1980 winter | 0.425462 | 0.387137 |
| winter1981 | 0.426437 | 0.435304 |
| winter1982 | 0.400673 | 0.405321 |

Table S4 Statistical results of life history parameters using 60 workflows with different bin of length (bin=10mm and bin=20mm), move average (MA) value (MA=5, MA=7, MA=9, MA=11) and four different methods workflows (ELEFAN_KS, ELEFAN_RSA, ELEFAN_SA, ELEFAN_GA).

| Reconstructed method of length frequency data | Workflows | *L_inf_* | *K* | *C* | *Rn_max_* |
| --- | --- | --- | --- | --- | --- |
| Bin=10mm, MA=7 | ELEFAN_KS | 430.47 | 0.51 | 0 | 0.233 |
|  | ELEFAN_RSA | 440.00 | 0.48 | 0 | 0.277 |
|  | ELEFAN_SA | 426.39 | 0.49 | 0.833 | 0.326 |
|  | ELEFAN_SA_V1 | 382.90 | 0.64 | 0 | 0.303 |
|  | ELEFAN_SA_V2 | 453.51 | 0.54 | 0 | 0.241 |
|  | ELEFAN_SA_V3 | 393.16 | 0.59 | 0 | 0.332 |
|  | ELEFAN_SA_V4 | 391.48 | 0.60 | 0 | 0.301 |
|  | ELEFAN_SA_V5 | 448.73 | 0.46 | 0 | 0.275 |
|  | ELEFAN_SA_V6 | 485.79 | 0.47 | 0 | 0.248 |
|  | ELEFAN_GA | 439.91 | 0.55 | 0.110 | 0.207 |
| Bin=10mm, MA=9 | ELEFAN_KS | 430.47 | 0.51 | 0 | 0.253 |
|  | ELEFAN_RSA | 372.00 | 0.35 | 0 | 0.332 |
|  | ELEFAN_SA | 466.10 | 0.41 | 0.901 | 0.465 |
|  | ELEFAN_SA_V1 | 372.05 | 0.35 | 0 | 0.369 |
|  | ELEFAN_SA_V2 | 367.08 | 0.37 | 0 | 0.308 |
|  | ELEFAN_SA_V3 | 448.24 | 0.45 | 0 | 0.415 |
|  | ELEFAN_SA_V4 | 371.69 | 0.36 | 0 | 0.366 |
|  | ELEFAN_SA_V5 | 380.43 | 0.35 | 0 | 0.375 |
|  | ELEFAN_SA_V6 | 376.80 | 0.40 | 0 | 0.293 |
|  | ELEFAN_GA | 416.56 | 0.51 | 0.817 | 0.467 |
| Bin=10mm, MA=11 | ELEFAN_KS | 430.47 | 0.35 | 0 | 0.278 |
|  | ELEFAN_RSA | 386.00 | 0.38 | 0 | 0.327 |
|  | ELEFAN_SA | 434.09 | 0.43 | 0.983 | 0.609 |
|  | ELEFAN_SA_V1 | 452.88 | 0.43 | 0 | 0.339 |
|  | ELEFAN_SA_V2 | 374.32 | 0.35 | 0 | 0.368 |
|  | ELEFAN_SA_V3 | 468.17 | 0.40 | 0 | 0.531 |
|  | ELEFAN_SA_V4 | 470.30 | 0.40 | 0 | 0.366 |
|  | ELEFAN_SA_V5 | 390.65 | 0.37 | 0 | 0.342 |
|  | ELEFAN_SA_V6 | 458.52 | 0.35 | 0 | 0.334 |
|  | ELEFAN_GA | 420.66 | 0.46 | 0.920 | 0.601 |
| Bin=20mm, MA=5 | ELEFAN_KS | 423.27 | 0.52 | 0 | 0.291 |
|  | ELEFAN_RSA | 381.00 | 0.61 | 0 | 0.306 |
|  | ELEFAN_SA | 379.70 | 0.65 | 0.657 | 0.438 |
|  | ELEFAN_SA_V1 | 440.58 | 0.48 | 0 | 0.302 |
|  | ELEFAN_SA_V2 | 364.10 | 0.63 | 0 | 0.250 |
|  | ELEFAN_SA_V3 | 375.98 | 0.60 | 0 | 0.401 |
|  | ELEFAN_SA_V4 | 352.89 | 0.37 | 0 | 0.444 |
|  | ELEFAN_SA_V5 | 455.06 | 0.45 | 0 | 0.387 |
|  | ELEFAN_SA_V6 | 507.51 | 0.60 | 0 | 0.285 |
|  | ELEFAN_GA | 415.34 | 0.53 | 0.825 | 0.297 |
| Bin=20mm, MA=7 | ELEFAN_KS | 423.27 | 0.51 | 0 | 0.268 |
|  | ELEFAN_RSA | 360.00 | 0.37 | 0 | 0.331 |
|  | ELEFAN_SA | 342.63 | 0.62 | 0.991 | 0.436 |
|  | ELEFAN_SA_V1 | 376.47 | 0.63 | 0 | 0.276 |
|  | ELEFAN_SA_V2 | 363.19 | 0.35 | 0 | 0.267 |
|  | ELEFAN_SA_V3 | 497.31 | 0.63 | 0 | 0.479 |
|  | ELEFAN_SA_V4 | 349.09 | 0.38 | 0 | 0.353 |
|  | ELEFAN_SA_V5 | 346.60 | 0.40 | 0 | 0.426 |
|  | ELEFAN_SA_V6 | 376.47 | 0.63 | 0 | 0.276 |
|  | ELEFAN_GA | 425.58 | 0.50 | 0.687 | 0.321 |
| Bin=20, MA=9 | ELEFAN_KS | 423.27 | 0.52 | 0 | 0.205 |
|  | ELEFAN_RSA | 366.00 | 0.35 | 0 | 0.244 |
|  | ELEFAN_SA | 481.21 | 0.38 | 0.998 | 0.440 |
|  | ELEFAN_SA_V1 | 358.71 | 0.36 | 0 | 0.249 |
|  | ELEFAN_SA_V2 | 508.71 | 0.45 | 0 | 0.286 |
|  | ELEFAN_SA_V3 | 501.27 | 0.62 | 0 | 0.382 |
|  | ELEFAN_SA_V4 | 353.84 | 0.37 | 0 | 0.258 |
|  | ELEFAN_SA_V5 | 435.24 | 0.49 | 0 | 0.300 |
|  | ELEFAN_SA_V6 | 365.52 | 0.35 | 0 | 0.247 |
|  | ELEFAN_GA | 427.87 | 0.51 | 0.711 | 0.404 |

ELEFAN_RSA: K-Scan for the estimation of K for a fixed value of L*_inf_*

ELEFAN_RSA: ELEFAN with response surface analysis

ELEFAN_SA: ELEFAN with simulated annealing

ELEFAN_SA_V1—V6: ELEFAN with simulated annealing with constrained t*_s_*=0 and C=0

ELEFAN_GA: ELEFAN with a genetic algorithm

L*_inf_*: asymptotic length

K: growth coefficient

C: intensity of seasonality

*R_n_*: a score value of goodness fit of growth curve

**REFERENCES**

Bivand, R. et al. 2020. classInt: Choose Univariate Class Intervals (R package version 0.4-3).

Caddy, J. F. et al. 1998. How Pervasive is “Fishing Down Marine Food Webs”? - Science 282: 1383–1383.

Chang, Y.-J. et al. 2013. Modelling the impacts of environmental variation on the habitat suitability of swordfish, *Xiphias gladius*, in the equatorial Atlantic Ocean. - ICES Journal of Marine Science 70: 1000–1012.

Chen, X. et al. 2009. Habitat suitability index of Chub mackerel (*Scomber japonicus*) from July to September in the East China Sea. - Journal of Oceanography 65: 93–102.

Gayanilo, T. C. et al. 1997. FAO - ICLARM stock assessment tools.

Lin, L. et al. 2004. Analysis of population biology of small yellow croaker *Pseudosciaena polyactis* in the East China Sea region. - Journal of Fishery Sciences of China (in Chinese with English abstract) 11: 333–338.

Mildenberger, T. K. et al. 2017. TropFishR: an R package for fisheries analysis with length-frequency data. - Methods in Ecology and Evolution 8: 1520–1527.

Pauly, D. 1983. Some simple methods for the assessment of tropical fish stocks.

Pauly, D. 1987. A review of the ELEFAN system for analysis of length-frequency data in fish and aquatic invertebrates. - In: Pauly D, Morgan R (eds), Length-Based Methods in Fisheries Research: ICLARM Conference Proceedings 13,468p. Manila: International Center for Living Aquatic Resources Management; Safat: Kuwait Institute for Scientific Research, pp. 7–34.

Pauly, D. 1990. Length-converted catch curves and the seasonal growth of fishes. - Fishbyte 8: 33–38.

Pauly, D. and David, N. 1981. Elefan I, a BASIC program for the objective extraction of growth-parameters from length-frequency data. - Kommission für Meeresforschung 28: 205–211.

Somers, I. F. 1988. On a seasonally oscillating growth function. - Fishbyte 6: 8–11.

Sparre, P. and Venema, S. C. 1998. Introduction to tropical fish stock assessment: Part 1 Manual. - FAO.

Then, A. Y. et al. 2015. Evaluating the predictive performance of empirical estimators of natural mortality rate using information on over 200 fish species. - ICES J. Mar. Sci. 72: 82–92.
